# Position-Sensitive Domain-by-Domain Switchable Ferroelectric Memristor

**DOI:** 10.1021/acsnano.4c14727

**Published:** 2025-02-13

**Authors:** Felix Risch, Panagiotis Koutsogiannis, Yuri Tikhonov, Anna G. Razumnaya, César Magén, José A. Pardo, Igor Lukyanchuk, Igor Stolichnov

**Affiliations:** † Nanoelectronic Devices Laboratory (NanoLab), Ecole Polytechnique Fédérale de Lausanne (EPFL), 1015 Lausanne, Switzerland; ‡ Instituto de Nanociencia y Materiales de Aragón (INMA), CSIC-Universidad de Zaragoza, 50009 Zaragoza, Spain; § Departamento de Física de la Materia Condensada, Universidad de Zaragoza, 50018 Zaragoza, Spain; ∥ Laboratory of Condensed Matter Physics, University of Picardie, 80039 Amiens, France; ⊥ Jozef Stefan Institute (JSI), Jamova Cesta 39, 1000 Ljubljana, Slovenia; # Departamento de Ciencia y Tecnología de Materiales y Fluidos, Universidad de Zaragoza, 50018 Zaragoza, Spain; ∇ Laboratorio de Microscopías Avanzadas, Universidad de Zaragoza, Campus Río Ebro, 50018 Zaragoza, Spain

**Keywords:** ferroelectrics, charged domain walls, memristor, multilevel memory, domain structure

## Abstract

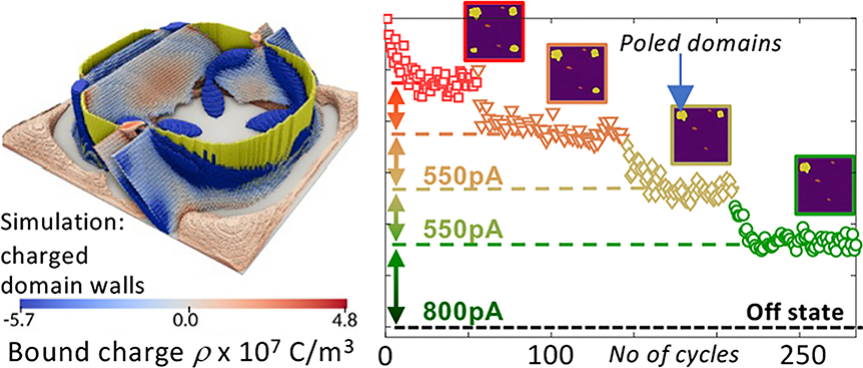

Domain-wall electronics based on the tunable transport in reconfigurable ferroic domain interfaces offer a promising platform for in-memory computing approaches and reprogrammable neuromorphic circuits. While conductive domain walls have been discovered in many materials, progress in the field is hindered by high-voltage operations, stability of the resistive states and limited control over the domain wall dynamics. Here, we show nonvolatile memristive functionalities based on precisely controllable conductive domain walls in tetragonal Pb(Zr,Ti)O_3_ thin films within a two-terminal parallel-plate capacitor geometry. Individual submicron domains can be manipulated selectively by position-sensitive low-voltage operations to address distinct resistive states with nanoampere-range conduction readout. Quantitative phase-field simulations reveal a complex pattern of interpenetrating a- and c-domain associated with the formation of 2D conducting layers at the intertwined regions and the emergence of 3D percolation channels of extraordinary high conductivity. Subnanometer resolution polarization mapping experimentally proves the existence of such extensive segments of charged tail-to-tail domain walls with unconventional structure at the ferroelastic-ferroelectric domain boundaries.

Conductive ferroelectric domain walls (DWs), two-dimensional entities forming conductive channels, which can be created, erased and manipulated upon request by electric fields have captured a wide interest in the field of nanoelectronics with the potential to be used in artificial synapses or as reconfigurable channels.^1−5^ The groundbreaking discovery of conductive DWs in BiFeO_3_ (BFO) by Seidel et al.^6^ was followed by reports of conductive DWs in a wide range of ferroelectric and multiferroic materials like Pb(Zr,Ti)O_3_^7^ (PZT), BaTiO_3_,^8^ LiNbO_3_^9^ (LNO), ErMnO_3_^10^ and others.^11−13^ Despite the strong promise of the concept, progress toward applications is hindered by low DW-conduction,^7,14,15^ complex poling or fabrication procedures^16,17^ or the volatility of charged DWs.^8,18^ The use of partially charged DWs permitted the realization of binary memory prototypes,^15,19−21^ in which the domain wall bridges two electrodes to enable a resistive switching. Further development resulted in proof of concept demonstrations of domain wall transistors^22,23^ and logic circuits^24,25^ as well as other application-relevant aspects including their technological integration,^23,24,26^ high-temperature stability,^27^ their network-behavior,^28^ domain wall *p*–*n* junctions^29^ and tunable in-plane DWs.^30^

One of the sought-after advanced functionalities, multiresistve state switching, can be achieved within the realm of DW-based ferroelectric systems in two ways: either by changing the conductivity of single DWs or by controlling the domain wall density. For single-DW tuning, various approaches were studied, including, e.g. changing of the surface angle in cone shaped DWs in LNO,^18^ modifying the proportion of the charged segments within DWs in BFO^31^ or dynamic tuning of the conductivity by consecutive pulses.^28,32^ Multidomain memristors, which rely on the stochastic process of polarization reversal involving many domains, address the issues of low current and variability of individual DWs.^33^ The reported LNO memristor of this type had a robust multistate conduction with a remarkably high number of discernible states, however a high operation voltage and large area of devices were required to fully exploit these benefits. Another persisting challenge impeding the exploitation of sophisticated DW-based devices is the reliable control over the domain wall dynamics. Attempts to manipulate the domain wall position include the creation of defects and pinning centers,^34,35^ inhomogeneous electric fields by the electrode shape,^15,36,37^ single-direction domain wall diodes^38^ or by stress induced formation and movement of new DWs.^12,39^ While showing that a DW control is possible, most of these approaches offer very limited flexibility due to a direct and irreversible change (mechanical, chemical, etc.) in either the electrode or interelectrode area.

In this work, we combine an advanced flexible control over the domain nucleation and propagation with stable and highly conductive DWs in a simple parallel-plate capacitor geometry resulting in a DW density memristor with multiple independently accessible and well-discriminated conduction states. The position-sensitive poling of individual submicron sized domains is achieved by utilizing high-resistive (high-R) top electrodes leading to nonvolatile multiresistive states that can be addressed repeatedly by selective domain-by-domain switching. The high and stable nA-range current outputs are mediated by partially charged boundaries of interconnected ferroelectric/ferroelastic domains in the tetragonal Pb(Zr,Ti)O_3_ films as revealed by a combination of quantitative phase-field modeling and experimental polarization mapping.

## Results and Discussion

### Confining Conductive 180°-DWs inside a Capacitor

**Figure 1:**
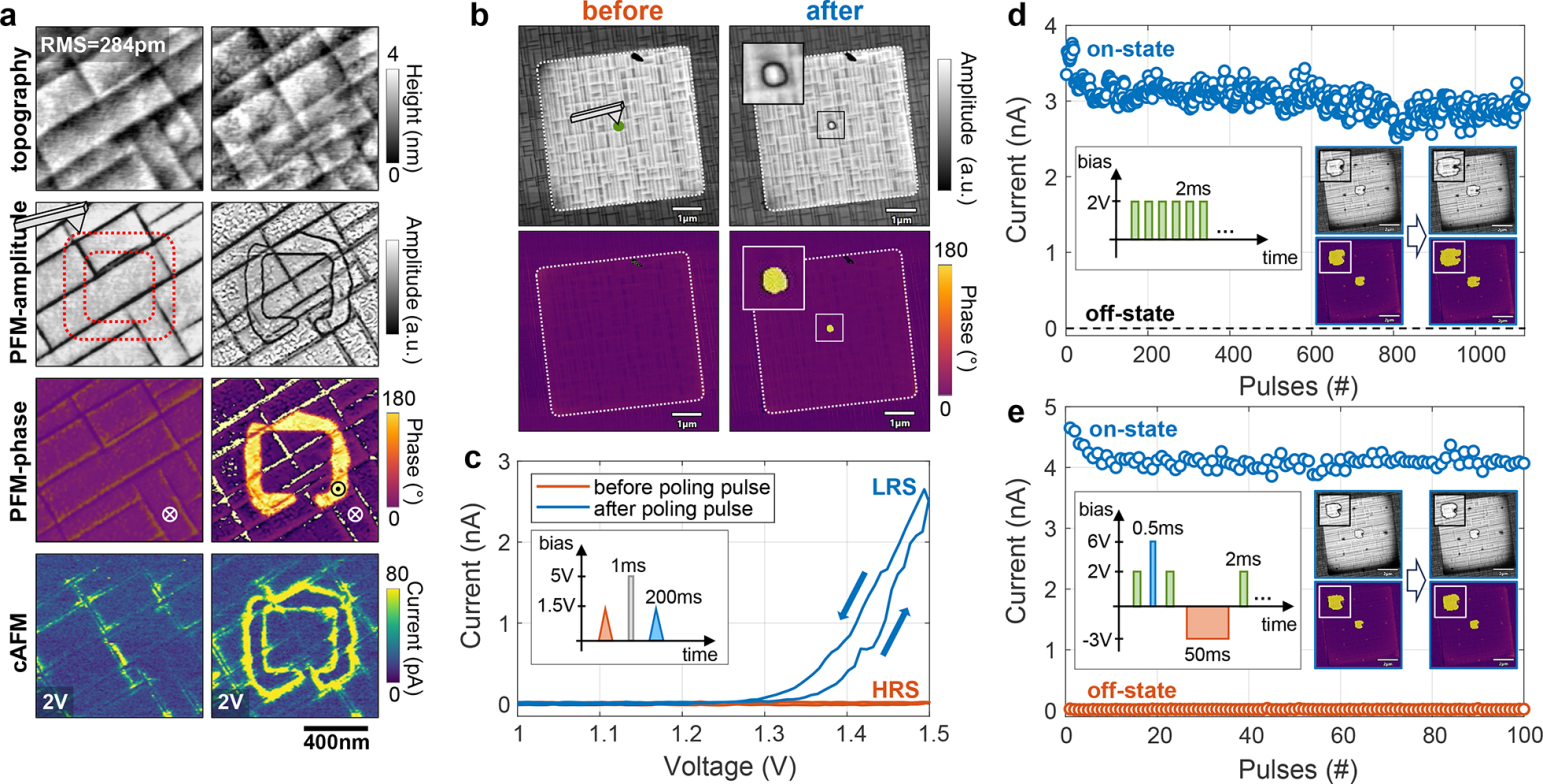
Ferroelectric domain wall properties and high-R Pt device characteristics. (a) From top to bottom: topography, PFM-amplitude, PFM-phase and cAFM images of the PZT bare surface. In the left column the pristine film is shown with uniformly downward polarized c-domains (purple) and weak 90°-DW conduction along the a-domain pattern (black lines of the cross-hatch pattern). In the right column, a square in a square poling was done indicated by the red dotted line. The poled segments shown in yellow, are bordered by conductive 180°-DWs. (b) PFM images of a high-R Pt electrode before and after a 5 V/1 ms poling pulse. A round polarized domain is observed after the poling together with a ring-like 180°-DW. (c) IV characteristics recorded by the AFM-tip at the same position before and after the poling (green dot). Without any 180°-DW, the conductance of the device is below the noise level of 5 pA (HRS). After the injection of the 180°-DW the conduction is increased to around 3 nA (LRS). (d) Retention test of the LRS. A circular domain is poled first and imaged by PFM, then 1100 1.5 V/2 ms pulses are applied and the current response is recorded. A PFM image is taken after the test to confirm the stability of the circular poled domain. (e) Endurance test by an alternating sequence of injecting and erasing of a polarized domain. After each operation the conductance state of the device is recorded by 1.5 V/2 ms pulses. PFM images before and after are taken to monitor the reproducibility of the poling process.

PZT (Zr/Ti = 10:90) films of 60 nm thickness were epitaxially grown by pulsed laser deposition (PLD) onto a (110) DyScO_3_ (DSO) substrate together with a 20 nm thick SrRuO_3_ (SRO) bottom electrode (details in Methods). Through PFM imaging the pristine downward polarized c-domains, which are intersected by ferroelastic a-domains, can be visualized (Figure 1a). Phase loops acquired with the AFM-tip on the surface yield a switching window of ∼±2 V (see Supporting Information). In these samples the 90°-DWs as well as the 180°-DWs, which are created by poling, exhibit a conductive response, which can be revealed by cAFM imaging (Figure 1a, bottom row).

In previous works^20,39,40^ the conduction properties of the 90°- and 180°-DWs were studied and basic memristive operations were demonstrated. However, by using Cr/Au evaporated electrodes as described in ref.,^20^ it was not possible to confine written 180°-DWs under the top electrode area. The demonstrated configurations used a DW-electrode connection which was obtained by pushing the 180°-DWs from outside of the device area to the boundary of the electrode to form binary on/off-switching devices. Device states in which the 180°-DW was positioned directly under the electrode were unstable and tended to collapse once electrical readout or consecutive PFM imaging was performed (see Supporting Information), preventing any precise DW-conduction tuning.

To enable a better and flexible control over the domain wall position inside the device area, electron beam induced deposited (EBID) platinum (Pt) top electrodes were employed (details in Methods). These electrodes possess a relatively high sheet resistance – up to 3 orders of magnitude higher than that of conventional electrodes of the same thickness.^41^ The elevated resistance limits the speed of charge movement, resulting in a voltage gradient across the high resistive (high-R) electrode when subject to sufficiently short voltage pulses.^42,43^ Therefore, by tuning the amplitude and dwell time of the poling pulses, the propagation of the DWs can be controlled enabling position-sensitive poling/reading operations. This approach allows to confine the polarization domains to submicron areas beneath the conductive probe inside the capacitor device. In the pulse mode, each individual domain can be selectively written and erased without impacting adjacent domains. Additionally, the high-R Pt electrodes facilitate the separation of nondestructive readout and write operations, allowing for domain-wall current sensing that does not significantly change the shape of the written domains.

In Figure 1b (left column) PFM images of a pristine 5 × 5 μm^2^ high-R Pt electrode with a thickness of 12 nm are shown. PFM amplitude and phase images show that the domain configuration and polarization state of the capacitor can be monitored through the top electrode. For manipulating the domain configuration, to inject or erase DWs and to probe the conductance state of the device, the AFM-tip is used as a nanometric contact. By placing the AFM-tip in the center of the electrode and after the application of a short voltage pulse (5 V/1 ms), a circular domain is formed under the tip’s location (right column of Figure 1b). The resulting conductance change of the device from its original high-resistive state (HRS) to its low-resistive state (LRS) can be observed in Figure 1c, in which IV curves from before (red) and after (blue) the poling pulse are recorded. In the pristine HRS without any 180°-domain wall, a current lower than the noise level of around 5 pA for 1.5 V is measured. After the poling pulse, the injected domain wall mediates a current transport providing a readout of ∼3 nA which results in an on/off ratio of at least 3 orders of magnitude. The on/off switching as well as the current readout are performed from the same location in the center of the electrode (green dot).

In Figure 1d,e, retention and endurance cycling tests are performed, respectively. For the retention test, a single circular domain is poled (insert PFM image, left side) and successively probed by rectangular 2 V/2 ms readout pulses. A total of 1100 pulses are recorded consecutively without degradation of the LRS. Subsequent PFM imaging (insert PFM image, right side) confirmed the stable domain structure. In the endurance test, a circular domain is alternately created and erased with readout pulses before and after each operation. The test performed over 100 cycles shows excellent current stability. PFM images before and after cycling (see PFM inserts) show nearly the same domain configuration confirming the stability and control of the injected and erased DWs. Subsequent tests showed that nondegrading cycling for more than 700 cycles can be performed with only slight variations of the LRS (see Supporting Information).

### From Binary to Multistate Memristor

**Figure 2:**
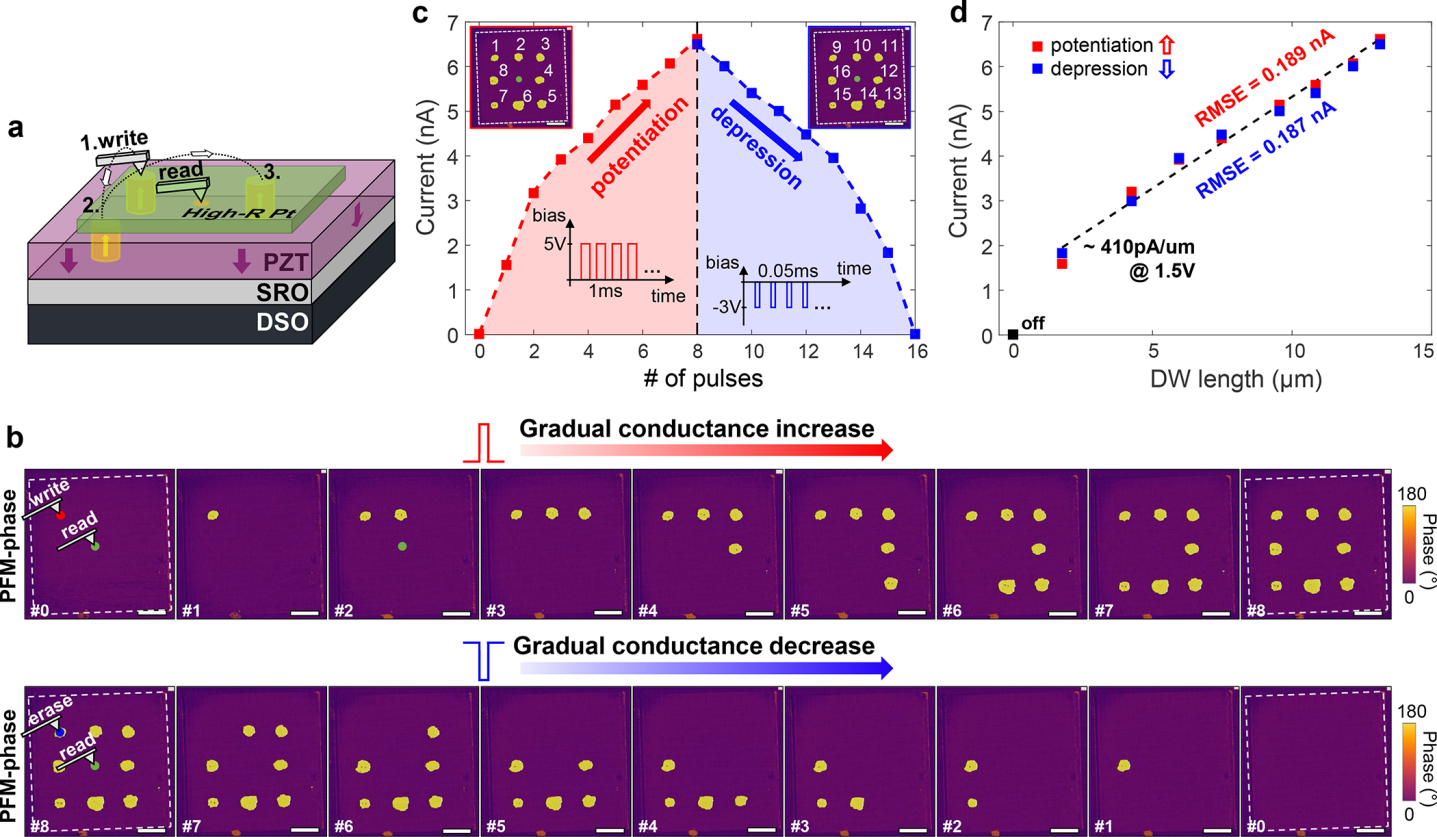
Multistate domain wall memristor. (a) Device schematic and poling operations. (b) PFM-images of each device state after consecutive poling operations. Top row shows the one-by-one addition of poled domains (yellow) by application of positive pulses. Bottom row shows the step-by-step turning off of individual domains by negative pulses. (c) Simultaneously recorded change of conduction for each of the 9 levels (8 poled domains + off state) from the experiment in (b). The red curve indicates the increase in conduction when more domains are poled and the blue curve corresponds to the case when domains are switched off and the conductance decreases. (d) Plot of the conduction values over the total domain wall length (DW-length acquired by calculating the accumulated domain circumference of each state). A linear behavior is apparent with a slope value of 410 pA/μm. A high degree of symmetry between the potentiation and depression curves is obvious and supported by the root-mean-square error (RMSE) values of 0.189 nA and 0.187 nA respectively.

These characteristics pave the way for more advanced multiresistive level device concepts, relying on repeatedly creating and erasing of multiple domains and a resulting step-like conductance change. In Figure 2a, the working principle of the device is illustrated. By varying the tip position on the high-R Pt electrode and the application of short (5 V/5 ms) voltage pulses an independent set of DWs can be injected in the device. In Figure 2b PFM images taken after each poling operation are shown, revealing the step-by-step creation of new domains (top row). Notably, the application of subsequent poling pulses does not lead to any visible change of the shape or position of the already existing domains due to the well confined electric field. By simultaneously recording IV curves after each operation (see Supporting Information), a gradual increase of the conductivity can be observed (red squares in Figure 2c). The IV curves are taken from the center of the device (green dot) and show a DW-mediated current up to a maximum of ∼6 nA for 8 created domain wall rings. Even more importantly it is possible to selectively turn off the switched domains again by adjusting the width and amplitude of the backpoling pulses (−3 V/80 μs). By placing the tip at the previously poled domain locations, a sequential deactivation of single domains is realized (bottom row of Figure 2b). Consequently, by erasing the DWs associated with the poled domains the conductivity of the device is gradually decreased (blue squares in Figure 2c). Moreover, the conduction profile (”potentiation/depression curve”) plotted over the number of created/erased domains is found to be nearly linear and fully symmetric, compared to similar pulse modulation schemes in memristive FeFET devices, which suffer from high nonlinearity and asymmetry.^44,45^

In Figure 2d, the potentiation and depression curves are plotted over the total DW-length of each device state (DW-length is measured as the accumulated circumference of the polarized domains), confirming the linear dependency of conduction over DW-length with a slope of around 410 pA/μm. The high symmetry between the potentiation and depression curve results from the high degree of control over the size of domains and their excellent reversible character. The high linearity is explained by the fact that each poling operation at a different location creates another low-resistive path for the current to pass the capacitor similar to a parallel resistor circuit. Moreover, by tuning of the poling pulses and reducing the thickness of the high-R Pt electrode it was possible to inject 5 domains in a sub 0.25 μm^2^ area without a collapse of the domain configuration and a single domain size of 50 nm (see Supporting Information), demonstrating the scalability of the approach.

### Repeatable Addressing of Multiple Resistive States

**Figure 3:**
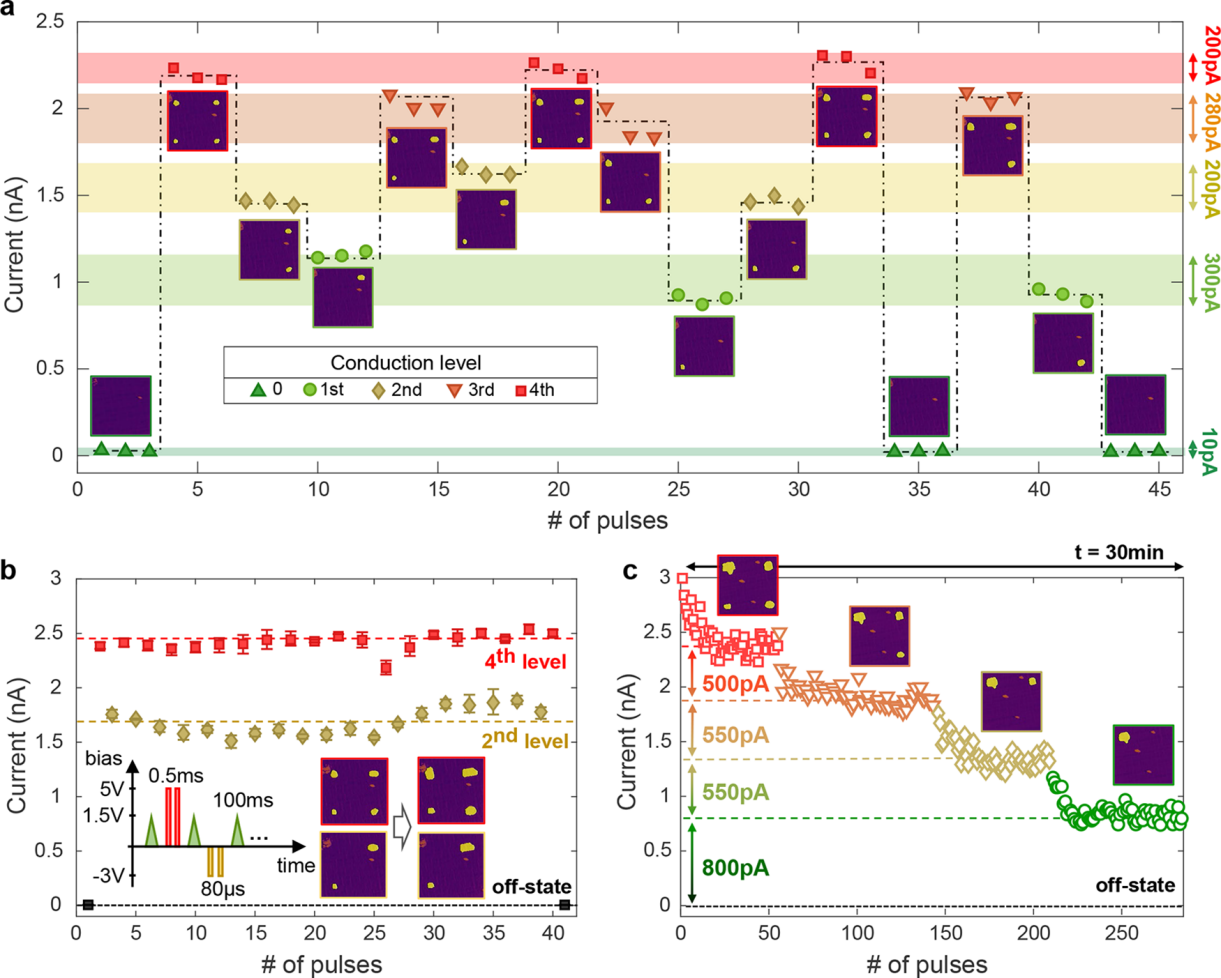
Repeatable multilevel addressing of nonvolatile conduction states. (a) Independent addressing of 4 conduction levels in a random sequence. Each conduction level is addressed 3 times and the parameters of the poling/depoling pulses were 5 V/0.5 ms and −2.5 V/80 μs, respectively. After each operation 3 readout pulses (1.5 V/100 ms) are recorded before switching to the next level and PFM images are taken after each step to confirm the correlation between conduction state and domain configuration. (b) Multilevel endurance test by cycling between the fourth and second conduction level of the same device as in (a). PFM images before and after the test (inserts) confirm the stability of the written domain patterns. (c) Retention characteristics of the 4 conduction levels, probed by consecutive application of around 70 pulses (1.5 V/100 ms). After a stabilization phase for around 5 pulses (∼1 s) the conduction remains stable and each level is well separated by at least 500 pA. PFM inserts show the domain configuration of each state.

To demonstrate the potential and robustness of this approach for a multistate memristor, a sequence is performed in which different conduction levels are addressed independently of the previous state and in a repeatable manner. For the data in Figure 3 a device similar to the one from Figure 2 is used with a 7 × 7 μm^2^ surface area. In Figure 3a, starting from the off-state with monodomain configuration and a current readout lower than the noise level of 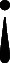
5 pA, the device is cycled through different domain configurations ranging from 0 to 4 poled domains. The domains are arranged in a square (see PFM-phase inserts at each conduction level) and are located around the readout point (center of the device). An irregular sequence is performed in which each conduction level is addressed 3 times. After applying the poling (5 V/0.5 ms) and depoling (−2.5 V/80 μs) pulses needed to reach the new state, the conduction is read from the center with 3 consecutive pulses (1.5 V/100 ms). Each resistive state could repeatably be addressed, meaning that after cycling through different configurations, it was possible to restore the conduction values of that level within a margin of 200–300 pA.

In Figure 3b, the multilevel endurance is tested by cycling between the conduction level corresponding to 2 and 4 poled domains. Between each readout pulse two poling/depoling pulses are applied, respectively. For up to 40 times, the domains were cycled and showed reasonable stability. For the second level an increase of around 100 pA after 28 cycles is observed, with the overall conduction being confined within a margin of 280 pA. During this fatigue test, some domains exhibit a tendency to grow after cycling due to the alternating poling pulses with higher amplitude than the readout pulses. Importantly, this domain expansion has minimal impact on the multilevel functionality. The current variation in Figure 3b remains within 10% without any consistent trend of increase across cycles. A plausible explanation is that domain wall conduction does not scale linearly with the domain circumference, and additional sections formed during domain expansion seem to contribute very weakly to conduction. Further explanations of this behavior are presented in the next section. The multistate fatigue test illustrates that repetitive cycling and measuring is possible not only for complete on/off-switching as shown in Figure 1, but also between different groups of domains. It is worth noting that the pulses used for cycling the system in the multibit storage mode (Figure 3b) are different compared to the single-bit switching in Figure 1e. Cycling between the 4-domain state and 2-domain state requires precise voltage pulse adjustments to selectively erase two domains while leaving the other two virtually unaffected. Pulses of −3 V/80 μs were determined to be sufficiently short to reproducibly remove the domain under the tip with the minimal impact to the adjacent domains. These pulses could also be applicable for the endurance test in Figure 1e, however in that case much longer −3 V/50 ms pulses were used. These long pulses ensure complete polarization reversal, effectively erasing small domain nuclei near the bottom interface, thereby subjecting the fatigue test to more rigorous conditions.

To complement these experiments, Figure 3c shows the retention of the 4 resistive levels used in Figure 3. For each level around 70 readout pulses (1.5 V/100 ms) are recorded. Between each 5 pulses a waiting time of several seconds is introduced to simultaneously test for a medium time-stability. Each level was probed for a total time of 2–3 min and the total experiment lasted for around 30 min (taking into account the time to switch levels and to acquire the PFM images for each state). A slight decrease for the first 3–5 pulses (∼1 s) of around 500 pA is observed which readily stabilizes. The stabilized discrete levels show a separation of ≥500 pA between each other with a scattering within one level of ≤300 pA. These observations further strengthen the nonvolatile character of each addressable conduction state with high robustness over repetitive readouts and longer time scales. The operation speed is another important characteristic of the memory element, which can be estimated from the data in Figure 3. The demonstrated writing/erasing speeds of 500 μs/80 μs (Figure 3b) are comparable to those of flash memories, albeit with lower voltage requirements. In the presented experiments, the switching speed is primarily constrained by extrinsic factors such as capacitance and electrode resistance. Further iterations of this concept with electrical contacts replacing the AFM tip and with a smaller capacitor size, are expected to yield significantly faster current responses.

### Modeling

To gain deeper insights into the switching mechanism between high- and low-resistivity states, we conducted phase-field modeling of a PZT thin film region with a thickness of 60 nm and periodically constrained lateral dimensions of 250 × 250 nm^2^ (details see Methods). In our simulation, we identified the bound charges, characterized by the divergence of the polarization field and concentrated with a density of ρ = −div **P**. These bound charges are screened by semiconducting free charges, typically originating from lattice imperfections and impurities such as oxygen vacancies.^39^ The free charges, attracted by the bound charges, form memristive channels. We demonstrate that the complex topological networking of the 180° and 90° DWs results in an intricate, percolating configuration of these channels. Notably, this is an inherently 3D effect of domain wall interlacement, which makes it challenging to identify on the 2D slices of the structure commonly used for the analysis of polarization patterns.

**Figure 4:**
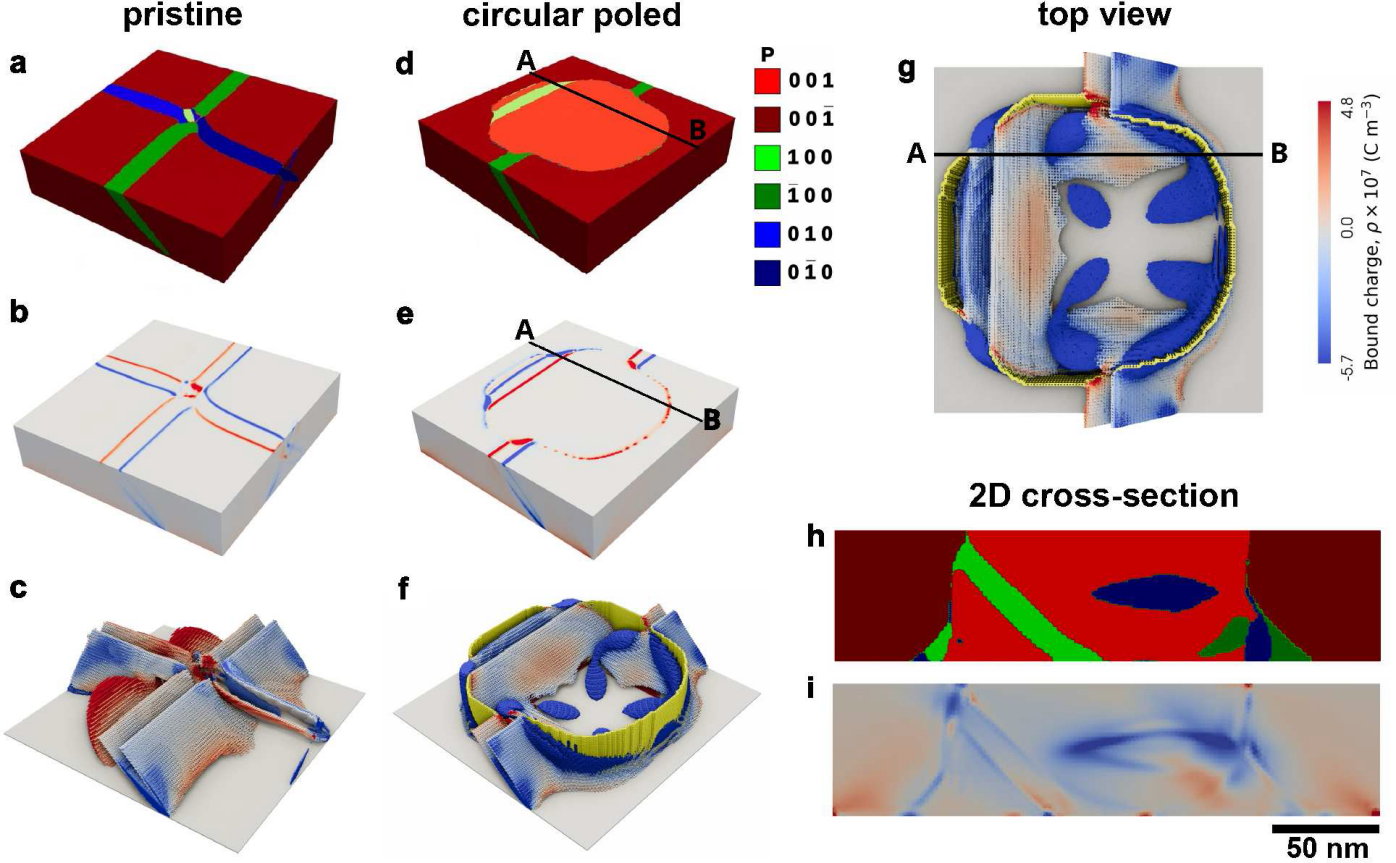
Domains, DWs and bound charges in PZT films: a phase-field simulation. (a) Polarization distribution at the surface of the pristine film with a- and c-type domains. The color map illustrating the polarization orientation is displayed at the top-center of the figure. (b) Bound charges at the termination of 90° DWs at the surface. (c) 3D phase-field tomography of the domain wall structure and bound charge distribution inside the pristine film. Gray inclined surfaces represent the 90° DWs. The positive and negative bound charges are shown in red and blue, respectively. (d) Polarization distribution at the surface of the film after poling and relaxation. (e) Distribution of the bound charge at the surface after poling and relaxation. (f) 3D phase-field tomography of the DW structure and bound charge distribution inside the film after poling and relaxation. The 180° DW is shown in yellow. (g) Top view of the 3D phase-field tomography image of panel (f) showing the extended areas of negative bound charges (blue) around the 180° DWs (yellow). (h) 2D cross-cut slice along the line AB at panel (d) showing the distribution of polarization. (i) 2D cross-cut slice along the line AB at panel (e) showing the distribution of bound charge.

We investigate the dynamics of polarization domains and bound charges that form the memristive channels within the film during the poling process. A cylindrical volume with a radius of *R* = 100 nm is poled by applying a bias −6 V at the surface. Panels a–c of Figure 4 illustrate the distribution of domains and charges before poling, while panels d-i show the distribution after poling when the voltage is removed and the system is relaxed.

Before poling, the sample is predominantly polarized downward along the c-direction (dark red color). The network of narrow a-type domains (blue and green color) emerges to compensate for the strain introduced by the interface with the DSO substrate. Two crossing a-type domains piercing the film are shown in Figure 4a. Figure 4b illustrates the surface emergence of bound charges, associated with 90° DWs of these domains. The origin of the charges is related to a slight deviation of the 90° walls from the charge-neutral 45° orientation. This configuration optimizes the elastic energy associated with matching a-type and c-type domains.^39^ These charges exhibit alternating, positive (red) and negative (blue) signs on opposite sides of the a-type domains, depending on the direction of polarization turn at the DWs.

The 3D phase-field tomography of the DWs and bound charges distribution beneath the surface, shown in Figure 4c, gives more information about the structure of the system. We observe that the regions with the highest concentration of bound charges are located at the 90° DWs near the interface with the substrate. This phenomenon is attributed to the additional deviation of the DWs from their 45° orientation to align with the substrate. Another notable feature is the emergence of a-type domains at the interface that do not extend to the upper surface, collapsing, instead, within the bulk of the c-phase. Due to their substantial curvature at the collapse points, these walls also host relatively large bound charges, that are mostly positive.

We now focus on the situation after poling, which reverses the polarization within the poled cylindrical volume. As illustrated in the surface view at Figure 4d, the majority of the poled area consists of upward-directed c-type domains (light red color). Most of the a-type domains have been displaced from the surface of the poled region. Only a small segment of an a-type domain, shown in green, remains within the poled area, near the border. Importantly, the surface-bound charges are now located not only along the DWs of this residual 90° domain, but also around the perimeter of the poled area, where the 180° domain wall emerges, marking the change in polarization of the out-of-plane direction (see Figure 4e).

Analysis of the full 3D phase-field tomography of the DWs and bound charges gives a comprehensive understanding of the bound charge distribution. The side and top views of the tomography are shown in Figure 4f,g, respectively. These images demonstrate the interlacement of the 180° (yellow color) and 90° DWs (gray color), resulting in the intricate distribution of bound charges which are heavily concentrated at the intersection points of the a- and c-type domain boundaries. Even more notable is the interaction between the 180° and 90° DWs at the sides of the poled region, where the 180° DWs are pierced by intersecting, mutually perpendicular 90° DWs, separating two variants of a-type domains.

Figure 4h,i, illustrates the domain and charge distributions beneath the surface along the vertical cross-section A-B, referenced in panels (d) and (e). These images provide useful information on the location of the bound charges within the bulk of the film. A spot of bound charges is observed in Figure 4i at the junction of the 90° and 180° DWs in the upper left corner of the poled area, just below the place of the surface emergence of the 180° domain wall, as shown in Figure 4h. This observation aligns with findings from our previous 2D simulations,^20^ which concluded that the observed conductivity of the 180° DWs are due to their networking with conducting 90° DWs. Another important observation is that the 180° DWs that bound the central c-domain exhibit a slight deviation from their equilibrium vertical orientation. This deviation becomes stronger as it approaches the interface with the substrate. Furthermore, a small portion of a-type domains is nucleated at the region where the domain wall meets the substrate. This effect, observed in both the left and right 180° DW is associated with the polarization bending near the substrate to accommodate the lattice matching between the ferroelectric material and the substrate. These deviations of the 180° DWs, which are typically charge-neutral in equilibrium, lead to the emergence of bound charges at the walls, and thereby providing their conductivity.

Another observation in Figure 4i is the nearly horizontal locus of bound charges at the central part of the cross-sectional area, which may also host a memristive channel. These charges originate from the intersection of the [01̅0] a-type domain (blue area in Figure 4h) and the [001] c-type domain (light red area in Figure 4h). However, the 2D slice does not provide complete information regarding the origin of the bound charge spots or their interconnectivity, which is needed to understand the formation of the memristive channels.

The complex arrangement of entangled a-type and c-type domains, which is fully revealed in the 3D view, leads to the formation of extended zones with predominantly negative bound charges (see Figure 4f,g), which is consistent with the formation of a 2D hole gas that supports domain wall conduction. Significantly, the conductive channels exhibit an intricate, continuous percolating distribution throughout the volume from bottom to top, a characteristic that can not be fully captured in 2D cross sections. These channels likely serve as current pathways within the memristor. Overall, the system reveals a complex network of interconnected conductive spots. The issue of conductivity becomes a percolation problem, where free charges navigate through these spots from the top to the bottom of the film. By tuning and switching the domain wall network with an electric field, the conductive channels within the volume can be rearranged, imparting the system with distinct memristive properties that are ideal for neuromorphic application.

### STEM-Cross Section Analysis of 180° Domain Walls

To confirm the emergence of the charged domain wall segments calculated by the phase-field modeling, we prepared a scanning transmission electron microscopy (STEM) specimen from one of the poled areas of a similarly produced PZT film (details in Methods). More specifically, ferroelectric domain switching was implemented in areas of 5 × 10 μm^2^ by applying a positive bias of +5 V at the SrRuO_3_ and scanning the grounded tip in successive rectangular regions oriented along the <100>_pc_ direction of the DyScO_3_ substrate. This switching created consecutive downward poled regions between the pristine upward polarized areas separated by the formed 180° DWs. A cross-sectional lamella oriented along <010>_pc_ was extracted from the poled region by a focused ion beam, to determine the location of the 180° DWs and analyze their polarization, which could reveal the presence of charged segments.

**Figure 5:**
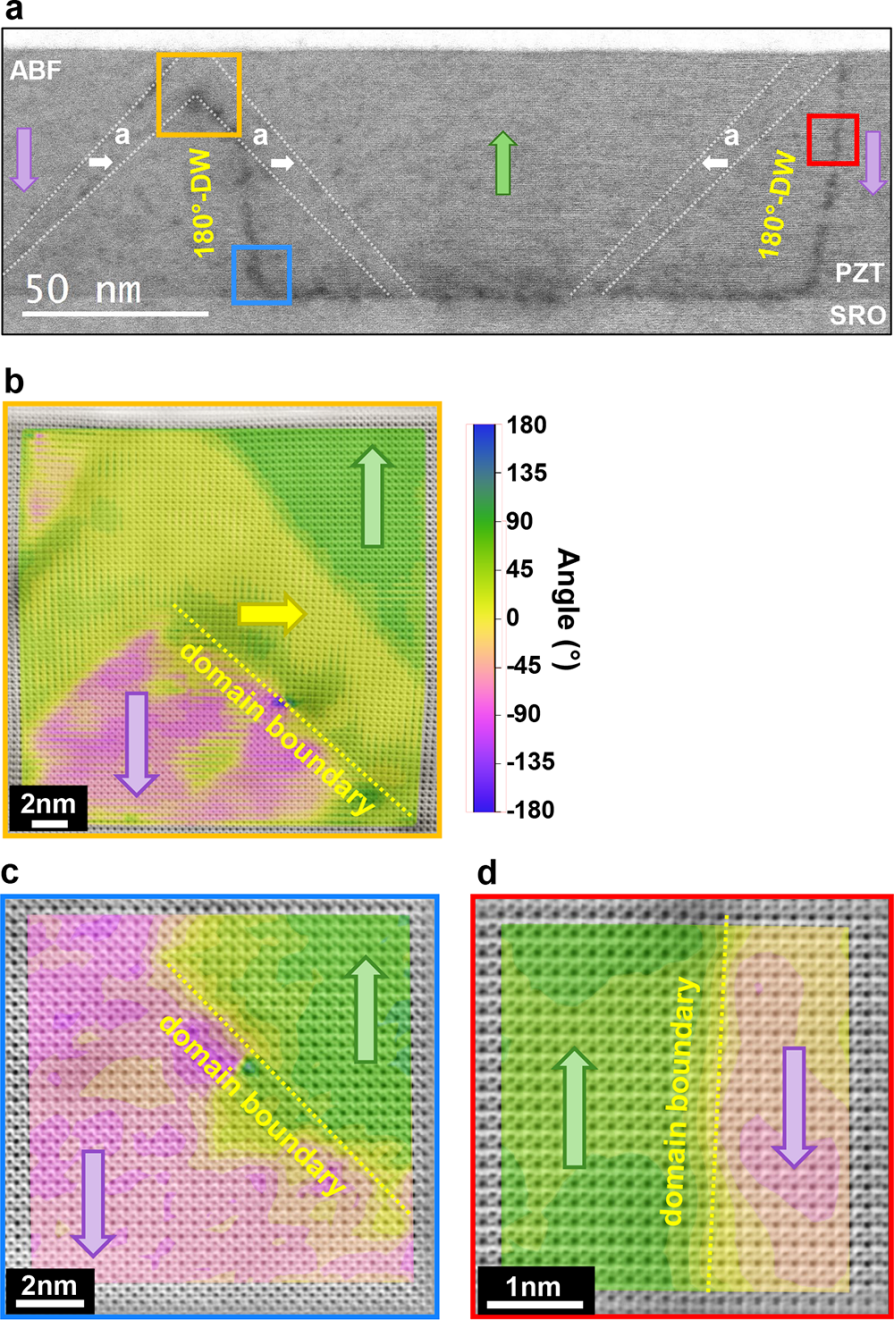
STEM images and polarization analysis of the PZT film and the 180° DWs. (a) Low-magnification ABF image of the film. The three a-domains are highlighted with dashed white dashed lines and the 180° DWs are identified by a black contrast. (b–d) High-resolution ABF images of the 180° domain wall at different locations collected from the yellow, blue and red square areas in (a). The images are overlaid with a color map of the analyzed polarization vector orientation highlighting the differently polarized domains and the domain boundaries. The polarization discontinuity at the domain boundary is highlighted with a yellow dotted line.

The 180° DWs locations within the film were located and imaged using an annular bright field (ABF) imaging. The 180° DWs, which form at the boundaries between the upward-poled domain and the two downward-poled domains, are shown in Figure 5a as dark-shaded regions. This shading stems from diffuse scattering caused by the shear strain that emerges at the defect following the poling process. In addition, the film is populated by a-type domains which are inclined along the (011)_pc_ and (01̅1)_pc_ planes of the tetragonal PZT.

The polarization of the domains was calculated by analyzing the positions of the Ti/Zr and O atoms. Specifically, the analysis showed that the c-domains of the as-grown film have an out-of-plane polarization pointing upward and the switched c-domains downward. The a-domain polarization is adapted to minimize the energy cost corresponding to the bound charges that emerge at the DWs between the c- and a-domains. To gain a deeper insight into the formation of the 180° DWs, we performed high-resolution ABF-STEM at the three designated areas shown in Figure 5a. The first image (highlighted in yellow, Figure 5b) is focused on the interconnection of the two a-domains. The image shows that the c-domain above the left a-domain and the c-domain beneath the two a-domains are switched and polarized downward. On the other hand, the c-domain above the right a-domain is polarized upward and therefore unswitched. The analysis of polar displacements reveals that both a-domains are polarized to the right, forming a polarization discontinuity at the boundary between the bottom domain wall of the right a-domain and the downward polarized c-domain which runs parallel to the (110)_pc_ plane. This discontinuity forms a tail-to-tail domain wall configuration which induces negative bound charges. Further down the film, the high-resolution ABF image near the bottom interface (highlighted in blue, Figure 5c) displays a 45° inclination of the 180° domain wall along the (110)_pc_ orientation before it reaches the substrate creating another charged segment with a tail-to-tail configuration. An explanation for this unexpected deviation from the charge neutral configuration could be that an a-domain previously existed in the region where the domain wall twist occurs but was eliminated due to the strong electric fields applied during poling. The residual stress left at the location of the former a-domain causes the 180° domain wall to twist and aligning it parallel to the (110)_pc_ plane. Finally, the 180° domain wall in the third image (highlighted in red, Figure 5d), which runs parallel to the (001)_pc_ plane and separates the two differently polarized c-domains, shows a slight bending confirming its weakly charged nature even in the absence of a-domain interactions.

Both inclined charged segments as well as the slightly bent 180° domain wall generate bound charges which attract screening charges from within the film and contribute to the conductivity of the formed domain wall channels. Interestingly, both predicted mechanisms (slight bending of the 180° DW and strongly charged tail-to-tail segments at the a-c domain boundary interaction points) from the phase field simulations are found in the 2D cross-section polarization STEM analysis, which support the proposed concept of a percolation mediated current transport through the 3D domain wall channels.

## Conclusions

To conclude, the integration of PZT films with highly conductive DWs and focused ion beam (FIB)-deposited electrodes presents a unique combination of properties, making them highly promising for multistate memristor applications. These films exhibit high DW conduction, achieving currents in the range of 1–10 nA/μm^2^ with the application of remarkably low voltages of just 2 V. The system supports multiple reproducible and randomly accessible states, as well as consistent, position-sensitive switching of individual domains. This innovative concept opens new pathways for research and development, particularly in integrating the memristor with access circuitry for individual domain read/write operations without relying on an AFM probe. Such circuitry could involve a network of high-conductivity leads connected to the high-resistance layer at specific points where domains are to be nucleated. With potential domain sizes as small as 10–20 nm, the design holds significant promise for scaling. However, challenges such as cross-talk between adjacent domains and achieving precise domain addressability require further investigation.

Compared to previously presented LNO based DW-memristors^33^ in which a high-density of electrically controllable domains allowed for the reproducible access to numerous conduction states, the memristor introduced in this work functions by manipulation of single DWs. It operates at much lower read/write voltages and may support more aggressive scaling by requiring fewer domains through a higher current over single domain wall ratio. However, these advantages come with trade-offs, including the need for more complex access circuitry and a reduced number of conduction states compared to the LNO-memristor.

The mechanism underlying the high domain wall conduction in tetragonal strained PZT films reveals a more intricate domain structure than previously thought. Phase-field simulations, supported by STEM atomic-scale analysis and polarization mapping, indicate that the interaction between DWs between the entangled a- and c-domains is central to the observed memristive properties. The simulations show the emergence of the complex network of bound charges, leading to the formation of an extended system of conductive channels percolating throughout the bulk of the film, and demonstrating that the enhanced conductivity is inherently a 3D volumetric effect. While conventional 2D analytical methods, such as STEM, have provided valuable insights and are able to support the proposed model, fully capturing the nature of the current transport in these systems necessitates a 3D reconstruction of the conductive channels.

Thus, this study demonstrates that highly conductive charged DWs can be effectively harnessed for information processing within well-established tetragonal perovskite ferroelectrics, which exhibit very stable and predictable domain structures.

## Methods

### Sample Fabrication

Highly tetragonal (Zr:Ti 10:90) 60 nm Pb(Zr,Ti)O_3_ (PZT) films were epitaxially grown onto (110) DyScO_3_ (DSO) substrates with a SrRuO_3_ (SRO) bottom electrode. DSO substrates (from CrysTec GmbH) with a miscut angle of 0.1° along the [01̅0] direction were treated before deposition by annealing and NaOH etching and annealing according to Kleibeuker et al.^46^ For the PLD deposition a 248 nm laser operated at 1 J/cm^2^ and the SRO layers were grown with 2 Hz at 625 °C and 0.145 mbar oxygen pressure. PZT films were grown with 3 Hz at 575 °C and 0.25 mbar oxygen pressure. Controlled cooling of the film was done with 1 mbar oxygen pressure at a rate of 15 °C/min. The film showed excellent flat surface quality, confirming good epitaxial growth conditions with a root-mean-square (RMS) value of the surface roughness of 287.4 pm (Figure 1a). The film thicknesses (60 nm/20 nm) for the epitaxial layers (PZT/SRO) were verified by the TEM cross-section analysis. The as grown uniformly downward polarized c-domains could be imaged and verified by the switching dynamics through PFM analysis.

### AFM Imaging and Electrical Characterization

All AFM/PFM/cAFM images were acquired by a commercially available Asylum Research ES Cypher AFM-system (from Oxford Instruments). The Cypher was equipped with the environmental control system (ES) to allow for precise temperature and Gas-environment control. Before imaging the samples were heated up for ∼10 min at 120–150 °C together with a flux of N_2_ to dehydrate the sample surface and environment. After cooling down the samples were kept and stabilized at room-temperature (306 ± 0.1 K) and a N_2_ rich environment was maintained. For all experiments conductive B-doped diamond coated tips from ADAMA Innovations (AD-40-AS) were used with a tip-radius of smaller than 10 nm and a stiffness of 4 N/m. Electrical readout and PFM poling/switching was performed using the mobile AFM tip to contact the top electrode, while the SRO bottom electrode (contacted by silver paste) was biased by the AFM controller. The current readout is performed by the Cypher Dual Gain ORCA Holder which is kept at virtual 0 V and all voltage biases are applied with respect to the SRO electrode.

### EBID Electrode Patterning

High resistive Platinum electrodes were deposited by a FEI Nova 600 NanoLab FIB tool using the electron beam operating at 5 kV and 1.6 nA. Individual pattern could be drawn and consecutively written by the electron beam by breaking of the organic Pt-rich organic precursor ((CH_3_)_3_CH_3_C_5_H_4_Pt). During the deposition carbon-rich organic defects are incorporated inside the electrode, which results in their high resistivity.

### STEM

Atomic characterization of a similarly produced PZT thin film was performed using a Thermo Fisher Scientific Titan Low Base 60–300 transmission electron microscope (TEM) operated at 300 kV. The used PZT sample for the STEM experiment possessed confirmed 180° and 90° domain wall conduction and only differed from the sample used for the AFM-experiments by the direction of the pristine c-domains (upward compared to the pristine downward oriented c-domains of the sample used for Figures 1–3). The TEM was equipped with a high-brightness Schottky field emission gun (X-FEG) and a Wien-filter monochromator. Sub-angstrom resolution in scanning transmission electron microscopy (STEM) mode was achieved using a CETCOR aberration corrector for the condenser system. An annular bright field (ABF) detector was used to highlight the contrast associated with the presence of DWs. Dedicated scripts were utilized to collect sets of 10 consecutive ABF images. These images were acquired with a short dwell time of less than 1 μs per pixel. The images were realigned with subpixel resolution and averaged to correct for residual spatial drift and reduce electronic noise.

The atomic displacements of Zr/Ti and O columns along the in-plane (Δ*x*) and out-of-plane (Δ*z*) directions relative to the substrate plane were calculated by measuring their shifts from the centrosymmetric positions defined by the A sites (Pb) of the perovskite structure. Initial positions of the atomic columns were identified using a blob detection algorithm that tracked intensity maxima, followed by refinement through successive center-of-mass and 2D Gaussian fits using the Atomap Python package.^47^ Detailed polar displacement maps were then constructed using the Temul package.^48^

Cross-sectional TEM lamellas were fabricated using a Thermo Fisher Scientific Helios 650 dual beam microscope. Samples were cut along the [100]pc crystal orientations of the DSO substrate.. The final thinning and polishing steps utilized low current (<7 pA) and voltage (5 kV) settings to reduce structural damage and prevent dislocation motion caused by heat and Ga+ ion bombardment.

### Phase-Field Modeling

#### Functional

Numerical simulations of conductive DWs in the ferroelectric PZT thin film were done through the minimization of the Ginzburg–Landau-Devonshire free energy functional^49^ for the pseudocubic ferroelectric material in which the elastic and electrostatic effects with account for free charge carriers are included:
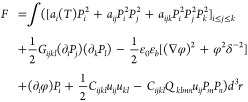
1Here the tensor summation notation over the repetitive indices that takes the Cartesian components *x*, *y*, *z* (or 1, 2, 3) is assumed.

Functional 1 includes the Ginzburg–Landau energy^50^ given in the first square brackets. The second term is the polarization gradient energy.^51^ Third and fourth terms represent the electrostatic energy, accounting also the screening effects.^52^ The last two terms correspond to the elastic energy. The electrostatic potential and strain tensor are denoted as φ and *u*_*ij*_ respectively. The value of the vacuum permittivity ε_0_ is 8.85 × 10^–12^ CV^–1^ m^–1^ and the value of the background dielectric constant ε_*b*_ is 10.^53^ The numerical values of the Ginzburg–Landau expansion coefficients *a*_*ijk*_, gradient energy coefficients *G*_*ijkl*_, elastic stiffness tensor *C*_*ijkl*_ and tensor of electrostrictive coefficients *Q*_*ijkl*_ are provided below.

The electrostatic properties of the system are described by the Poisson equation, ε_0_ε_*b*_ ∇^2^φ = −(ρ_bound_ + ρ_free_), that is governed by two types of charges. The density of the bound charges, ρ_bound_ = −div **P**_tot_, is provided by the nonuniform distribution of the total polarization in the bent 90°-DWs, which includes the spontaneous and field-induced parts: **P**_tot_ = **P** + (ε_*b*_ – 1)∇φ. Here ε_0_ is the vacuum dielectric permittivity and ε_*b*_ ≈ 10 is the background dielectric constant of nonpolar ions.^53^ The density of the uncompensated free charges is given by the linearized Thomas-Fermi equation ρ_free_ = −(ε_0_ ε_*b*_ δ^2^)^−1^φ. The screening length δ can be estimated^54^ through the Bohr radius *a*_0_ = 0.053 nm and the concentration of carriers *n*_0_ ≈ 10^20^ m^–3^ as  1.6 nm. This value is used in the phase-field calculations.

The distribution of the electrostatic potential φ and the elastic strains *u*_*ij*_ is found from the respective electrostatic (with screening) and elastic equations:
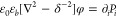
2
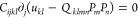
3

#### Material Coefficients

The coefficients of the Ginzburg–Landau expansion for Pb_0.9_Zr_0.1_TiO_3_ at room temperature^55^ are as follows: *a*_1_ = −0.1618 × 10^5^ C^–^^2^ m^2^ N, *a*_11_ = 0.3883 × 10^9^ C^–^^4^ m^6^ N, *a*_12_ = 0.6357 × 10^9^ C^–^^4^ m^6^ N, *a*_111_ = 0.2518 × 10^9^ C^–^^6^ m^10^ N, *a*_112_ = 0.8099 × 10^9^ C^–^^6^ m^10^ N, *a*_123_ = −4.3588 × 10^9^ C^–^^6^ m^10^ N (the second order coefficients *a*_*ij*_ are taken for the zero-strained sample. They are calculated from the stress-free coefficients, using the standard procedure^56^). The values of the electrostrictive tensor coefficients are *Q*_1111_ = 0.085 C^–^^2^ m^4^, *Q*_1122_ = −0.0251 C^–^^2^ m^4^, *Q*_1212_ = 0.0328 C^–^^2^ m^4^.^55^ Components of the elastic stiffness are *C*_1111_ = 1.7 × 10^11^ m^–2^ N, *C*_1122_ = 0.76 × 10^11^ m^–2^ N, *C*_1212_ = 0.83 × 10^11^ m^–2^ N. The Gradient energy coefficients^57^ are *G*_1111_ = 2.77 × 10^–10^ C^–^^2^ m^4^ N, *G*_1122_ = 0, *G*_1212_ = 1.38 × 10^–10^ bC^–2^ m^4^ N.

#### Phase-Field Modeling

The minimum of the free-energy is found as the solution of nonlinear differential relaxation eq 1:
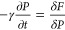
4Here γ is a time-scale parameter which is taken to be equal unity. The nonlinear part of the equations is closed by two linear systems of equations defined by the Poisson equation with screening 2 and the equation of linear elasticity 3.

The phase-field simulations were conducted using the FEniCS software package.^58^ Three-dimensional rectangular computational regions are represented by structured tetrahedral finite element meshes, that were created with the 3D mesh generator gmsh.^59^ The solutions for **P**, φ and *u*_*ij*_ was sought in the functional space of first order Lagrange polynomials.

The initial quenching from the paraelectric state was conducted with the imposition of Dirichlet boundary conditions at the bottom side of the computational region φ_bot_ = 0 and φ_top_ = 1 × 10^–6^ V at the top side. The application of the tip was simulated by imposition of the Dirichlet boundary condition φ = −6 V in the circular area of the tip application and zero everywhere else at the top side of the computational region. Substrate-induced strain is taken into account by imposition of the Dirichlet boundary conditions on components of the displacement vector **u** at the bottom surface of the thin film, *u*_*x*_ = *u*_0_*x*/*L*_*x*_ and *u*_*y*_ = *u*_0_*y*/*L*_*y*_, where *u*_0_ = 0.35% is the strain value, *L*_*x*_ = 250 nm and *L*_*y*_ = 250 nm are in-plane geometrical dimensions of thin film. Variables **P** and φ are constrained with periodic boundary conditions in the *x* and *y* directions.

The time derivative on the left-hand side of eq 4 is approximated by BDF2 variable time scheme.^60^ The paraelectric phase at the first-time step is a random distribution of the polarization vector components in the range of −10^–6^ to 10^–6^ Cm^–^^2^. Nonlinear system arising from eq 4 is solved by Newton method with line search. To solve the linear system on each nonlinear iteration and systems defined by eqs 2 and 3, the generalized minimal residual method with restart is used.^61,62^

## Data Availability

The data in this work is available from the authors upon reasonable request.

## Supporting Information Available

Piezoelectric loop of amplitude and phase of local piezoresponse probed by the AFM-tip on the surface of the PZT film, positioning a 180°-domain wall under a Cr/Au top-electrode, long-time endurance test of the memristor with high-resistance Pt electrode, *IV*-curves of multidomain memristive switching, and downscaling of polarization domains in the memristor with high-resistance Pt electrode (PDF)
