# Supplementary material for: Position-Sensitive Domain-by-Domain Switchable Ferroelectric Memristor
Source: ACS Nano. 2025 Feb 13;19(7):6993–7004. doi: 10.1021/acsnano.4c14727 (PMC11867021; doi:10.1021/acsnano.4c14727)
Supplement: Supplementary file 1 — nn4c14727_si_001.pdf [file nn4c14727_si_001.pdf]

ACS Nano

Supplementary Information

Position-sensitive Domain-by-Domain Switchable Ferroelectric Memristor

Felix Risch[1], Panagiotis Koutsogiannis[23], Yuri Tikhonov[6], Anna G. Razumnaya[7], César Magén[23], José A. Pardo[245], Igor Lukyanchuk[6], and Igor Stolichnov[1]

[1]Nanoelectronic Devices Laboratory (NanoLab), Ecole Polytechnique Fédérale de Lausanne (EPFL), 1015, Lausanne, Switzerland

[2]Instituto de Nanociencia y Materiales de Aragón (INMA), CSIC-Universidad de Zaragoza, 50009 Zaragoza, Spain

[3]Departamento de Física de la Materia Condensada, Universidad de Zaragoza, 50018 Zaragoza, Spain

[4]Departamento de Ciencia y Tecnología de Materiales y Fluidos, Universidad de Zaragoza, 50018 Zaragoza, Spain

[5]Laboratorio de Microscopías Avanzadas, Universidad de Zaragoza, Campus Río Ebro, 50018 Zaragoza, Spain

[6]Laboratory of Condensed Matter Physics, University of Picardie, 80039, Amiens, France

[7]Jozef Stefan Institute (JSI), Jamova Cesta 39, 1000 Ljubljana, Slovenia

Correspondence should be sent to igor.stolitchnov@epfl.ch

List of Supplementary information:

Supplementary Figure 1 | Piezoelectric phase loop on bare surface PZT film

Supplementary Figure 2 | Positioning a 180°-DW under a Cr/Au top-electrode

Supplementary Figure 3 | Long-time endurance test of high-R Pt memristor

Supplementary Figure 4 | IV-curves of multi-domain memristive switching

Supplementary Figure 5 | Downscaling of polarization domains under high-R Pt electrode

Supplementary Figure 1 | Piezoelectric phase loop on bare surface PZT film

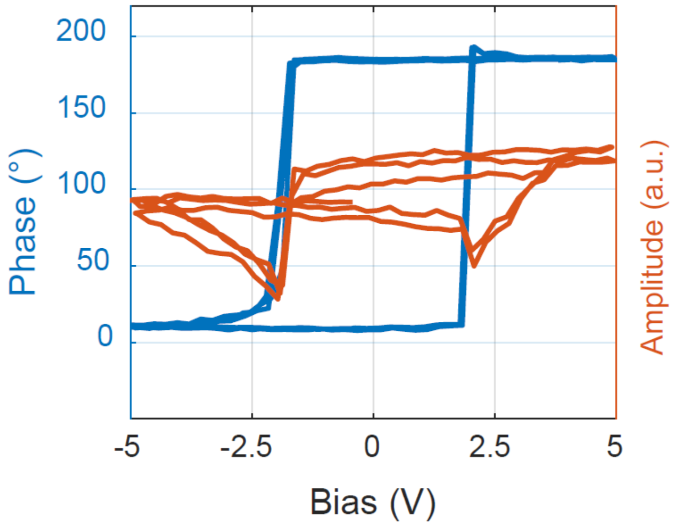

Piezoelectric loop of amplitude and phase of local piezo response probed by the AFM-tip on the bare surface of the PZT film. Data acquired with the DART mode (resonance enhanced mode) of the Cypher AFM.

Supplementary Figure 2 | Positioning a 180°-DW under a Cr/Au top-electrode

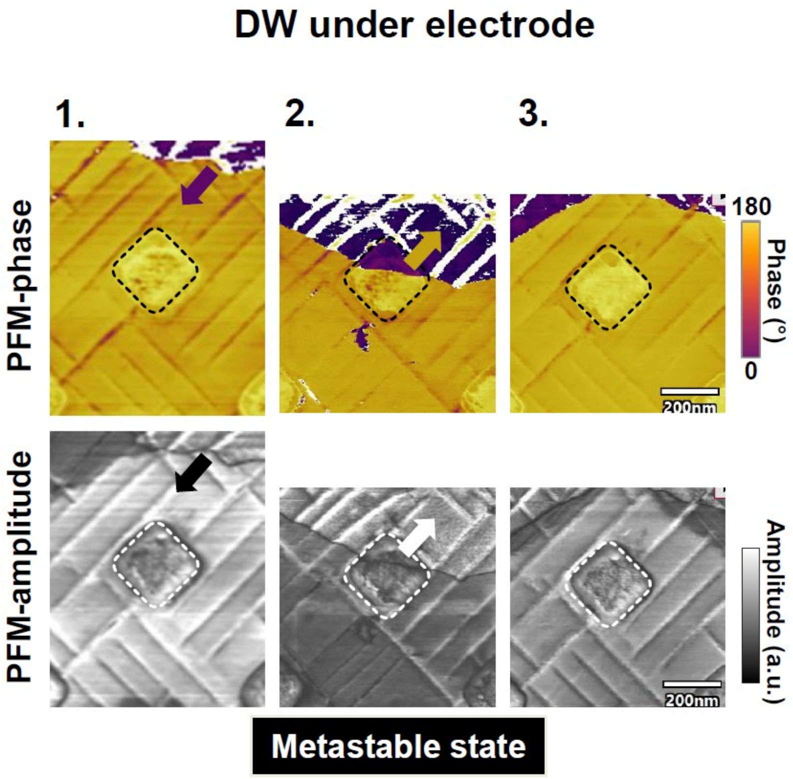

A 180°-DW is positioned under a square ~250x250 nm Cr/Au electrode (black/white dotted outlines in PFM images). In the top row the phase images of the PFM images are shown and the amplitude images in the bottom row.

From left to right:

1. Initially the whole area underneath the electrode is uniformly poled (yellow) and no 180°-DW is inside the device area.
2. By placing the AFM tip outside (but close to) the electrode boundary and applying a switching voltage, an extended area around the AFM-tip position is poled. The 180°-DWs, which outline the poled area are successively pushed underneath the electrode and inside the device area.
3. After trying to reading out the resistive state of the device from #2 by IV curves and trying to reimage the device by PFM, the loss of the device state from #2 is observed. It is concluded through more similar experiments that the creation of a state seen in #2 is first of all non-trivial as it difficult to replicate and secondly that once electrical (and mechanical pressure) stimuli is applied, the c-domains under the electrode area favor a uniformly polarized state. Hence no control over the DW position under the electrode area can be applied nor any device characteristics are possible to extract from such a metastable state.

Supplementary Figure 3 | Long-time endurance test of high-R Pt memristor

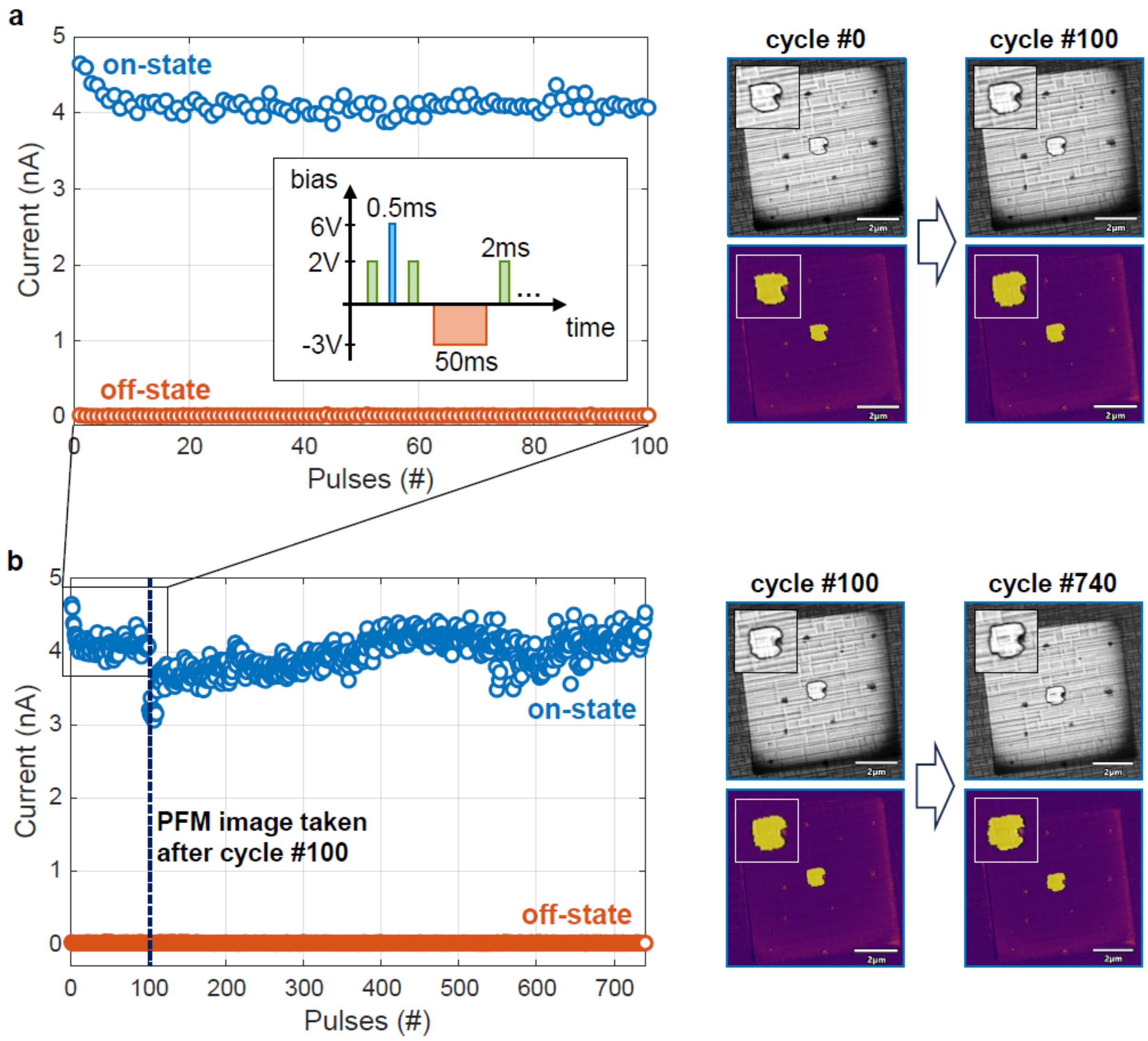

Supplement Figure 3a, shows the data as the Figure 1e from the main manuscript.

In Sup. Fig 3b, an extension of the endurance test in which the device is cycled between the HRS and LRS is shown. While some smaller distortions are evolving over the total of more than 700 cycles, the main characteristics of the LRS can be preserved and the before-after PFM comparison shows the near identical poling of the circular domain in the center of the device. The sharp decrease which is readily recovered after a few cycles, seen at cycle #100 is due to the PFM image taken after 100 cycles. Most probably scanning in contact mode, needed for PFM imaging slightly alters the tip-surface contact which results is the seen variations of the current readout.

Supplementary Figure 4 | IV-curves of multi-domain memristive switching

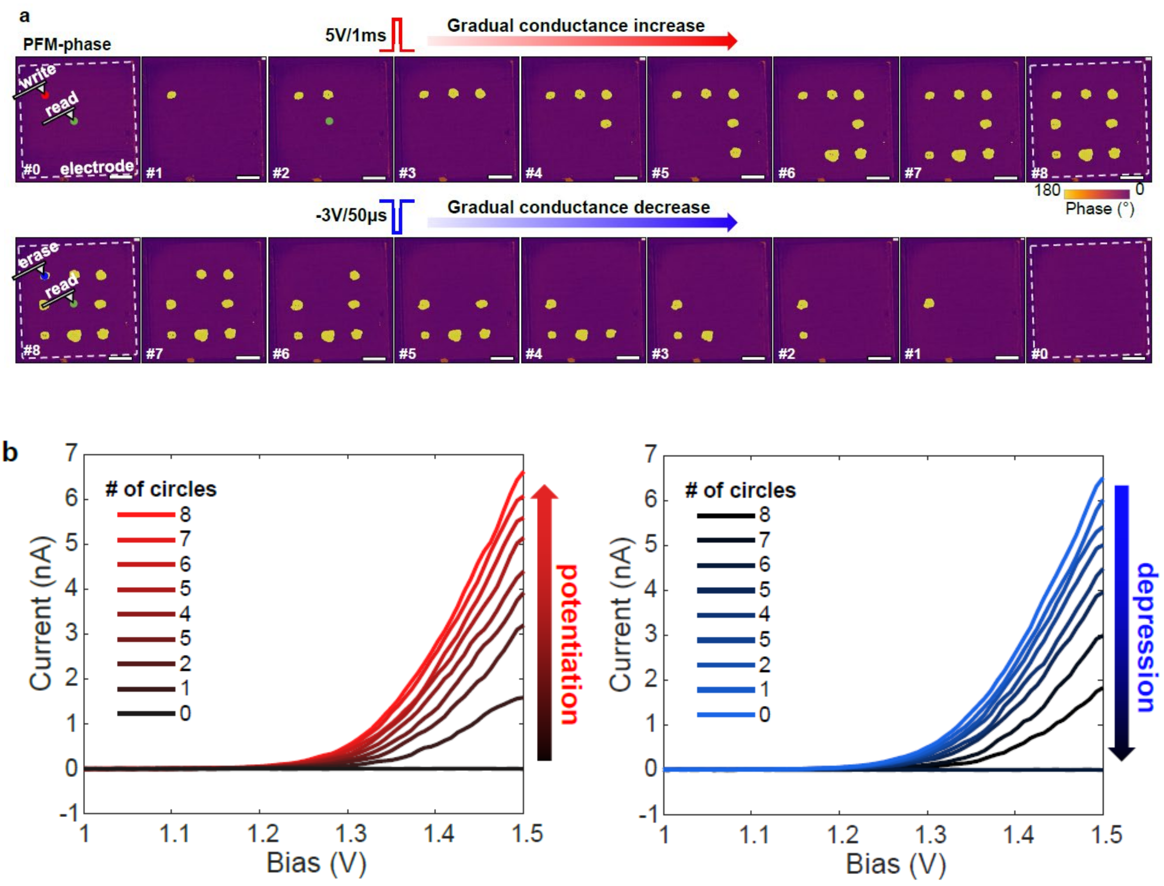

In Sup. Fig. 4b, the IV curves taken after each poling/repolling step are shown. The data corresponds to the experiment from the main manuscript of Figure 2 (Fig. 2b is shown here again for clarification as Sup. Fig. 4a). The IV curves are taken by placing the AFM-tip in the center of the device (green dot).

The maximum values of the IV curves at 1.5V from Sup. Fig. 4b are furthermore used for the analysis of the conduction to DW-length dependency of Figure 2c from the main manuscript.

Supplementary Figure 5 | Downscaling of polarization domains under high-R Pt electrode

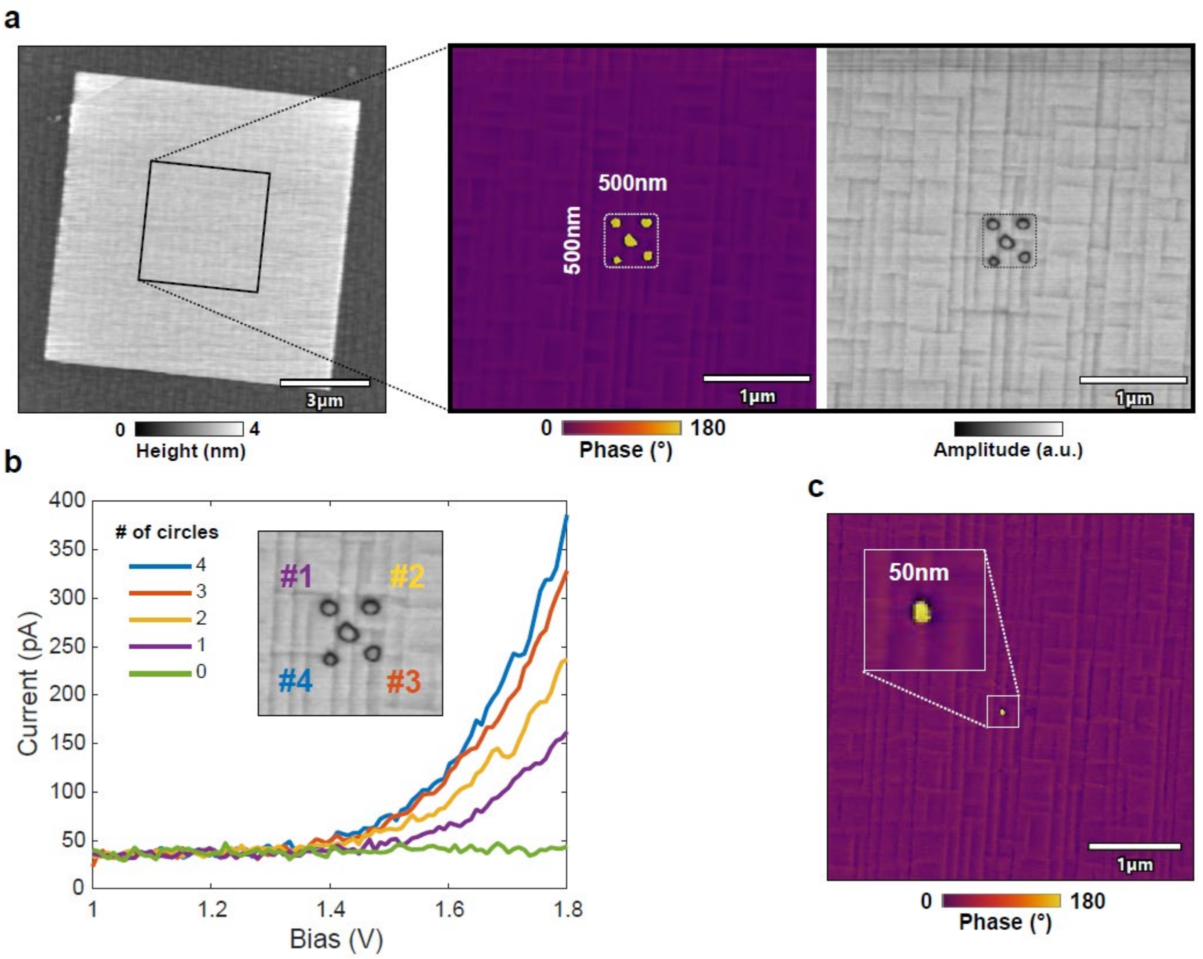

Sup. Fig. 5 showcases the downscaling possibilities of the polarization domains while using the high-resistive Pt top electrodes.

Compared to the high-R Pt electrodes from the main manuscript (with a thickness of 12 nm), the electrodes shown above are around 6 nm thick. The reduced thickness of the electrodes increase their sheet resistance which enables the further confinement of the applied electric-field pulses. Pulses of 5V/1ms are used to write circular domains with a minimum diameter of 50 nm. It was possible to write a set of 5 domains in a sub 0.25µm^2 area without destroying their structure and simultaneously reading out the non-volatile multi-resistive state characteristics. The reduced absolute current values resulted from the combination of overall reduced total DW-length + increased sheet resistance of the electrode material.

Together with other works regarding the lateral downscaling of the high-resistive electrodes (Ref: Van Dorp et al., *Approaching the resolution limit of nanometer-scale electron beam-induced deposition*. Nano letters 5, 1303–1307 (2005)), these results show the readily available possibilities of sub-µm downscaling of the proposed memristive devices.
